# Strength Development and Strain Localization Behavior of Cemented Paste Backfills Using Portland Cement and Fly Ash

**DOI:** 10.3390/ma12203282

**Published:** 2019-10-09

**Authors:** Yue Zhao, Abbas Taheri, Amin Soltani, Murat Karakus, An Deng

**Affiliations:** 1 School of Civil, Environmental and Mining Engineering, The University of Adelaide, Adelaide, SA 5005, Australia; Yue.Zhao@adelaide.edu.au (Y.Z.); Murat.Karakus@adelaide.edu.au (M.K.); An.Deng@adelaide.edu.au (A.D.); 2 Department of Infrastructure Engineering, Melbourne School of Engineering, The University of Melbourne, Parkville, VIC 3010, Australia

**Keywords:** cemented paste backfill, Portland cement, fly ash, curing time, unconfined compressive strength, digital image correlation, strain localization

## Abstract

This study examines the combined performance of Portland cement (PC), the binder, and fly ash (FA), the additive, towards improving the mechanical performance of the South Australian copper-gold underground mine cemented paste backfill (CPB) system. A series of unconfined compressive strength (UCS) tests were carried out on various mix designs to evaluate the effects of binder and/or additive contents, as well as curing time, on the CPB’s strength, stiffness and toughness. Moreover, the failure patterns of the tested samples were investigated by means of the three-dimensional digital image correlation (DIC) technique. Making use of several virtual extensometers, the state of axial and lateral strain localization was also investigated in the pre- and post-peak regimes. The greater the PC content and/or the longer the curing period, the higher the developed strength, stiffness and toughness. The use of FA alongside PC led to further strength and stiffness improvements by way of inducing secondary pozzolanic reactions. Common strength criteria for CPBs were considered to assess the applicability of the tested mix designs; with regards to stope stability, 4% PC + 3% FA was found to satisfy the minimum 700 kPa threshold, and thus was deemed as the optimum choice. As opposed to external measurement devices, the DIC technique was found to provide strain measurements free from bedding errors. The developed field of axial and lateral strains indicated that strain localization initiates in the pre-peak regime at around 80% of the UCS. The greater the PC (or PC + FA) content, and more importantly the longer the curing period, the closer the axial stress level required to initiate localization to the UCS, thus emulating the failure mechanism of quasi-brittle materials such as rock and concrete. Finally, with an increase in curing time, the difference between strain values at the localized and non-localized zones became less significant in the pre-peak regime and more pronounced in the post-peak regime.

## 1. Introduction

Mine tailings are among the largest and most problematic sources of solid waste, owing to their extensive production, durability over time, and potential health hazards—approximately 14 billion tons of tailings were produced globally by the mining industry in 2010 [1]. As of late, the concept of “sustainable mining” has gained increased attention; it can be defined as a set of engineering practices which effectively maintain a perfect balance between infrastructure performance and the social, economic and ecological processes required to maintain human equity, diversity and the functionality of natural systems [2]. Consequently, the transition towards sustainable mining warrants incorporating/reusing mine wastes, particularly tailings, as a “controlled low-strength material” (CLSM) in conventional infrastructure systems.

In its simplest terms, a CLSM can be defined as a high-density slurry composed of soil (mainly sands and/or silts), a cementitious binder (mainly Portland cement), and water; depending on the intended application, the slurry may be thickened to obtain a desired rheological behavior to accommodate facile pumping and effective field implementation. In this context, recent studies have reported innovative solutions to utilize mine tailings as an “additive” in the production of cement clinker, concrete and ceramic products; such approaches, which are becoming routine in practice, have promoted the sustainability of the mining industry [3,4,5]. Mine tailings prepared as CLSMs are self-compacting and flowable in character, and thus can be employed as a sustainable replacement for conventional structural fillings (e.g., backfilling of mined voids, bridge abutments, pipeline beddings and subbases in pavements) [6,7].

Cemented paste backfill (CPB) is a simple CLSM system composed of dewatered tailings, a cementitious binder and processed mine water; it is often thickened to obtain a non-settling character for facile pumping into mined cavities resulting from underground mine operations [8,9,10,11]. The desired rheological behavior, the non-settling character, often emerges at a solids content (by total mass) of 70%–85% [12,13,14]. In essence, CPB technology recycles tailings into underground mine excavations, and thus reduces the volume of surface-disposed tailings, mitigates the burden on the environment and assists waste management [10,15,16,17,18]. Over the past two decades, several systematic studies have been carried out to identify the governing variables which influence the mechanical performance (mainly strength and stiffness) of CPB systems [19,20,21,22,23,24,25]. This includes the physical, chemical and mineralogical properties of the tailings, the chemical composition of the mixing water, binder type (and its content), the adopted tailings–binder–water mix design (or solids content) and the in-situ stress and curing conditions. Although the effects of these variables have been well understood and documented in the research literature, the reported results are still not consistent, as the physical properties, chemical composition and mineralogical background of the tailings often varies from mine to mine. Accordingly, CPB projects are mainly characterized as site-specific, requiring the application of standard test methods, along with fundamental analysis and design procedures, to develop an acceptable design scheme.

According to the compressive damage zone (CDZ) crack framework, the failure process in quasi-brittle materials, and hence potentially CPBs, under compressive loading takes place within a so-called “local damage zone” (LDZ), where distributed cracking accompanied by a single major crack (i.e., single shear failure) or multiple major cracks (i.e., splitting failure) take place [26,27,28]. In this framework, the stress–strain response in the pre-peak regime describes the compressive behavior of the material in the entire sample [29]. Upon achieving the peak stress, accumulated large deformations take place (during unloading) outside of the damage zone; this is referred to as strain localization [30,31,32]. Quite clearly, a comprehensive understanding of the strain localization behavior of CPBs will be beneficial towards evaluating its damage evolution and failure mechanism under real-life loading regimes. To the authors’ knowledge, however, there are still no studies addressing the strain localization behavior of CPBs, and as such, further research is urgently required.

In view of the above, this study investigates the strength development and strain localization behavior of the South Australian copper-gold underground mine CPB system through a comprehensive experimental program. For this purpose, ordinary Portland cement (PC), the main cementitious binder, and fly ash (FA), the sustainable pozzolan additive, were employed. A series of unconfined compressive strength (UCS) tests were carried out on various PC + FA mix designs to evaluate the effects of binder and/or additive contents, as well as curing time, on the strength, stiffness and toughness of the CPB system. Common strength criteria for CPBs were also considered to assess the suitability of the tested mix designs for real-life applications. Moreover, an accurate non-contact strain measurement scheme—by means of the three-dimensional digital image correlation (DIC) technique—was implemented to: (i) measure the full-field of strain development on the surface of CPB samples; and (ii) characterize the deformations and strains of CPB samples with and without strain localization. Making use of several virtual extensometers, the state of strain localization was also investigated in both the axial and lateral directions.

## 2. Materials and Methods

### 2.1. Mine Tailings

A large quantity of processed mine tailings, sourced from a copper-gold underground mine in South Australia, was used in the present study [11]. The geotechnical properties of the tailings were determined in accordance with relevant ASTM (American Society for Testing and Materials) and Australian standards, and the results are tabulated in Table 1. In terms of grain-size distribution, the tailings consisted of 39% fines (<75 μm) and 61% sand (0.075–2 mm). The liquid limit (LL)—as determined for 20-mm cone penetration depth using the 80 g–30° fall-cone device—and standard thread-rolling plastic limit (PL) were measured as LL = 19.2% and PL = 13.1%, respectively; thus, a plasticity index (PI = LL − PL) of 6.1% was produced, such that the fines fraction of the tailings was classified as clay–silt with low plasticity (CL–ML) in accordance with the Unified Soil Classification System (USCS). The standard Proctor compaction test indicated an optimum water content of 8.7%, along with a maximum dry density of 2.06 g/cm^3^. The specific gravity of the tailings was found to be *G*_s_ = 2.61, which is slightly lower compared with that reported for natural CL–ML soils [33]. The chemical composition of the tailings, as supplied by the distributor, was mainly dominated by silicon dioxide (SiO_2_) and ferric oxide (Fe_2_O_3_), with mass fractions of 38.27% and 37.70%, respectively (see Table 1).

### 2.2. Binder, Additive and Mine Water

Ordinary PC often serves as a benchmark binding agent with respect to underground mine backfilling applications [11]. To effectively simulate the actual conditions of the South Australian copper-gold underground mine, a large quantity of PC was sourced from a local manufacturer and used as the main cementitious binder for CPB preparations. Its physical and chemical properties, as supplied by the manufacturer, are presented in Table 2. Moreover, standard Class C FA, was used as the siliceous–aluminous pozzolan additive to explore the possibility of partially replacing the main cementitious binder (or PC), thereby obtaining a more sustainable CPB design. The chemical composition of FA, as supplied by the manufacturer, was mainly dominated by silicon dioxide (SiO_2_), calcium oxide (CaO) and aluminum trioxide (Al_2_O_3_), with mass fractions of 40.2%, 24.3% and 18.7%, respectively.

A large amount of processed mine water, collected from the same copper-gold underground mine, was used for CPB preparations. The chemical composition of the mine water, as supplied by the distributor, is presented in Table 3. The chemical composition was mainly dominated by chloride (Cl^−^), sodium (Na^+^) and sulfate (SO_4_^2−^) ions, with concentrations of 5800, 3800 and 2400 mg/L, respectively. Other chemical properties included a pH of 7.5, such that the mine water was characterized as a neutral substance.

### 2.3. Mix Designs and Sample Preparations

A total of 20 PC + FA mix designs, as outlined in Table 4, were examined in this study. For ease of presentation, the following coding system is used to designate the various mix designs:

(1) $$P_{a}F_{b}T_{c}$$

where *P_a_* = *a*% PC; *F_b_* = *b*% FA; and *T_c_* = *c* days of curing.

The binder (PC or PC + FA), solids and water contents were all mass-based; they were, respectively, defined as:

(2) $$(\%)\;\mathrm{BC}=\frac{M_{B}}{M_{T}+M_{B}}\times 100$$

(3) $$(\%)\;\mathrm{SC}=\frac{M_{T}+M_{B}}{M_{T}+M_{B}+M_{W}}\times 100$$

(4) $$(\%)\;\mathrm{WC}=\frac{M_{W}}{M_{T}+M_{B}}\times 100$$

where BC = binder content (PC or PC + FA); SC = solids content; WC = water content; *M*_T_ = mass of tailings; *M*_B_ = mass of binder (PC or PC + FA); and *M*_W_ = mass of processed mine water.

Some reports indicate that the cost of CPB implementation tends to vary from 10% to 20% of the mine’s total operating cost, and the main cementitious binder, or PC, represents up to 75% of the total CPB cost [11]. In this regard, South Australian mining standards recommend a maximum PC content of 5% (by total dry mass). Accordingly, in this study, the tested PC contents ranged from 1% to 5% (see Table 4).

The tailings and binder (i.e., PC or PC + FA), were blended in dry form in accordance with the selected mix designs outlined in Table 4. Mixing was carried out for approximately 5 min to gain visible homogeneity of the ingredients. The required amount of mine water corresponding to a water content of WC = 30%—equivalent to a solids content of SC = 77%—was added to each blend and thoroughly mixed by a mechanical mixer to obtain slurries of uniform consistency. It should be noted that the choice of WC = 30% (or SC = 77%) was selected in accordance with South Australian mining standards; this water content produces the desired rheological behavior to accommodate facile pumping of the paste into mined cavities [11]. The resultant slurries were poured into cylindrical molds in one-third height increments; each layer was tamped 25 times using a small metal rod to remove entrapped air. The molds were then transferred to a humidity chamber, maintained at 70% relative humidity and a temperature of 25 °C, where curing was allowed for 14 days prior to testing. For those mix designs containing 4% PC, additional curing periods of 28 and 56 days were also considered to examine the effects of curing time on the CPB’s stress–strain attributes. It should be noted that the cylindrical molds measured 100 mm in height and 42 mm in diameter, thus producing a height-to-diameter ratio of 2.38 for the prepared samples, which is on par with that commonly adopted in the research literature [8,11,19,20,25].

### 2.4. UCS Test

**Figure 1:**
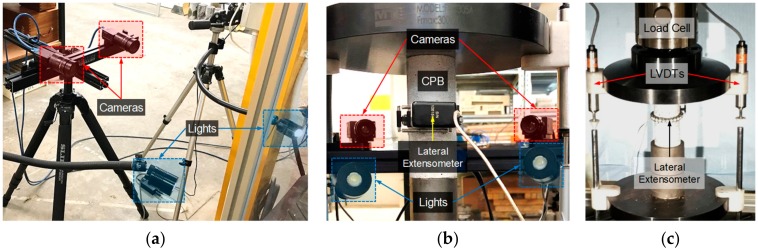
UCS + DIC testing system: (**a**) Two-camera stereo set-up; (**b**) Rear view of the experimental set-up during compressive loading; and (**c**) Front view of the experimental set-up during compressive loading.

The prepared 42-mm diameter by 100-mm long samples were subjected to the UCS test, as per ASTM C39–18, by means of a closed-loop servo-controlled hydraulic compressive machine with a maximum load capacity of 250 kN using the Instron-1342 model (Instron^®^, Norwood, MA, USA). Upon demolding, the two ends of the prepared samples were covered with a thin layer of dental paste to minimize any possible stress concentration, and to ensure surface homogeneity and hence uniform load distribution during compressive loading [34]. The prepared samples were axially compressed at a constant displacement rate of 0.1 mm/min. Axial deformations and the corresponding axial forces were digitally recorded by a real-time data acquisition system; the former was recorded using two external linear variable differential transducers (LVDTs), while the latter, the axial force, was measured by means of an external load cell (see Figure 1c). Moreover, a direct-contact lateral extensometer—632.12F20-series (MTS Systems Corp., Eden Prairie, MN, USA)—mounted at mid-length of the samples provided a signal for lateral deformations (see Figure 1b,c).

### 2.5. Three-Dimensional DIC Technique

DIC refers to a group of non-contact methods which acquire images of an object, store the images in digital form and perform image processing to extract full-field shape, deformation and motion measurements [35]. The present study specifically focuses on employing the three-dimensional DIC technique to characterize the relationships between strain localization and the macro-scale mechanical deformations of PC + FA CPB samples. In its simplest terms, the three-dimensional DIC technique is based on the calculation of surface deformations using a number of digital images acquired from a reference undeformed and subsequent deformed states [35,36]. The method involves the use of two high-resolution digital cameras positioned in such a manner that the surface patterns of the sample, undergoing compressive loading, can be captured from two different angles; in this manner, a three-dimensional measurement of the sample’s shape and displacements can be created.

As demonstrated in Figure 1a,b, the two digital cameras—positioned on the left and right sides of the desired sample—were symmetrically focused on the sample to capture digital grey-scale images during the UCS test. The used DIC set-up consisted of two Fujinon HF75SA-1 (Fujifilm Holdings Corp., Tokyo, Japan) monochrome cameras; camera specifications included an iris range of F1.4–F22, a focal length of 75 mm and a resolution of 5 megapixels. Moreover, uniform illumination was provided by two adjustable goose-neck halogen lights to ensure adequate contrast across the sample’s surface during imaging (see Figure 1a,b). The images were captured automatically using the VIC-Snap software package (Correlated Solutions Inc., Irmo, SC, USA). Prior to the UCS tests, each of the two cameras was stereo calibrated using a 30-mm standard target having a uniformly-spaced marker grid; calibration of the cameras was carried out by taking a minimum of 30 image-pairs at the calibration target [29]. During the UCS tests, the cameras were programmed to capture images automatically at a frame rate of one picture per second. For each of the tested samples, the first image-pair was defined as the undeformed state and served as a reference for the VIC-3D software package (Correlated Solutions Inc., Irmo, SC, USA) to recreate the deformation field on the subsequent image-pairs by means of the software’s built-in image processing algorithms.

## 3. Results and Discussion

### 3.1. Effect of PC + FA on UCS

**Figure 2:**
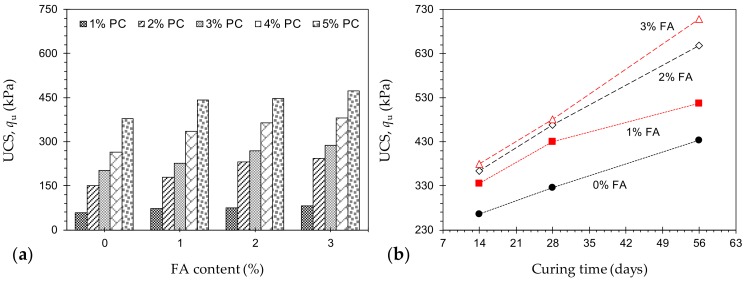
(**a**) Variations of UCS against FA content for various mix designs tested at 14 days of curing—*P_a_F_b_T*_14_ where *a* = {1, 2, 3, 4, 5}, and *b* = {0, 1, 2, 3}; and (**b**) Variations of UCS against curing time for various mix designs containing 4% PC—*P*_4_*F_b_T_c_* where *b* = {0, 1, 2, 3}, and *c* = {14, 28, 56}.

Figure 2a illustrates the variations of UCS against FA content for various mix designs tested at 14 days of curing—*P_a_F_b_T*_14_ where *a* = {1, 2, 3, 4, 5}, and *b* = {0, 1, 2, 3}. For any given FA content, the greater the PC content, the higher the developed UCS, following a monotonically-increasing trend. As typical cases, for FA = 2%, the use of 1%, 2%, 3%, 4% and 5% PC resulted in UCS values of *q*_u_ = 75.3, 231.7, 268.8, 363.5 and 446.7 kPa, respectively. Similarly, for any given PC content, an increase in FA content promoted a notable, yet less pronounced increase in the UCS. For instance, the samples *P*_4_*F*_0_*T*_14_, *P*_4_*F*_1_*T*_14_, *P*_4_*F*_2_*T*_14_ and *P*_4_*F*_3_*T*_14_ resulted in *q*_u_ = 265.3, 335.5, 363.5 and 379.7 kPa, respectively. Figure 2b illustrates the variations of UCS against curing time for various mix designs containing 4% PC—*P*_4_*F_b_T_c_* where *b* = {0, 1, 2, 3}, and *c* = {14, 28, 56}. For any given FA content, an increase in curing time promoted a major increase in the UCS, following a monotonically-increasing trend, thus signifying a time-dependent amending mechanism (i.e., pozzolanic reactions) [2,11,37,38,39,40]. The sample *P*_4_*F*_2_*T*_14_, for instance, resulted in *q*_u_ = 363.5 kPa, while the same mix design cured for 28 and 56 days led to higher values of 468.3 kPa and 648.3 kPa, respectively.

Common strength criteria for CPBs used in mining applications are summarized in Table 5. As is evident from Figure 2, the UCS for various PC + FA mix designs ranges between 59.7 kPa (for *P*_1_*F*_0_*T*_14_) and 708.5 kPa (for *P*_4_*F*_3_*T*_56_), thus indicating that none of the tested samples meet the prerequisite for roof support applications (i.e., *q*_u_ > 4000 kPa). The sample *P*_4_*F*_3_*T*_56_ resulted in *q*_u_ = 708.5 kPa, and thus satisfies the minimum 700 kPa threshold suggested for stope stability. For surface disposal applications, as well as general construction practices, all mix designs, excluding *P*_4_*F*_0_*T*_14_, *P*_4_*F*_1_*T*_14_, *P*_4_*F*_0_*T*_28_ and *P_a_F_b_T*_14_ (i.e., *a* = {1, 2, 3}, and *b* = {0, 1, 2, 3}), well meet the *q*_u_ ≥ 345 kPa criterion. Finally, all samples, excluding *P*_1_*F_b_T*_14_ (i.e., *b* = {0, 1, 2, 3}), satisfy the requirement for eliminating liquefaction in underground disposal applications.

**Figure 3:**
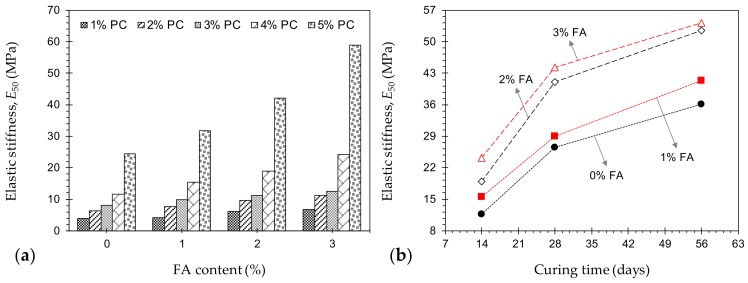
(**a**) Variations of *E*_50_ against FA content for various mix designs tested at 14 days of curing—*P_a_F_b_T*_14_ where *a* = {1, 2, 3, 4, 5}, and *b* = {0, 1, 2, 3}; and (**b**) Variations of *E*_50_ against curing time for various mix designs containing 4% PC—*P*_4_*F_b_T_c_* where *b* = {0, 1, 2, 3}, and *c* = {14, 28, 56}.

The secant modulus at 50% of the UCS, denoted as *E*_50_, serves as a measure of the material’s stiffness in the elastic compression domain [2,47,48]. The variations of *E*_50_, as outlined in Figure 3, exhibited a trend similar to that observed for the UCS; in general, for any given FA content, the greater the PC content, the higher the developed stiffness, following a monotonically-increasing trend. As typical cases, the samples *P*_1_*F*_2_*T*_14_, *P*_2_*F*_2_*T*_14_, *P*_3_*F*_2_*T*_14_, *P*_4_*F*_2_*T*_14_ and *P*_5_*F*_2_*T*_14_ resulted in *E*_50_ = 6.2, 9.7, 11.2, 18.9 and 42.2 MPa, respectively. Moreover, for any given PC content, an increase in FA content led to a notable, yet less pronounced increase in *E*_50_. For instance, the sample *P*_4_*F*_0_*T*_14_ resulted in *E*_50_ = 11.7 MPa, while the same mix design treated with 1%, 2% and 3% FA promoted higher values of 15.5, 18.9 and 24.2 MPa, respectively. Much like the UCS, the effect of curing time was found to be positively-proportional to the sample’s stiffness. The sample *P*_4_*F*_2_*T*_14_, for instance, resulted in *E*_50_ = 18.9 MPa, while the same mix design cured for 28 and 56 days promoted higher values of 41.1 and 52.5 MPa, respectively.

**Figure 4:**
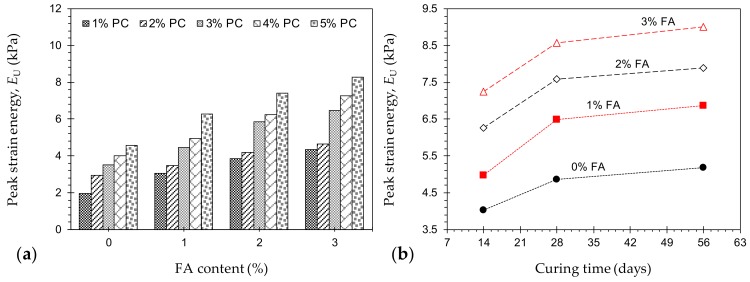
(**a**) Variations of *E*_U_ against FA content for various mix designs tested at 14 days of curing—*P_a_F_b_T*_14_ where *a* = {1, 2, 3, 4, 5}, and *b* = {0, 1, 2, 3}; and (**b**) Variations of *E*_U_ against curing time for various mix designs containing 4% PC—*P*_4_*F_b_T_c_* where *b* = {0, 1, 2, 3}, and *c* = {14, 28, 56}.

The area under a typical axial stress–axial strain curve (i.e., axial strain refers to that obtained by the external LVDTs, as shown in Figure 1c) up to the peak point (or the UCS) signifies the energy stored by a sample undergoing deformation, and thus serves as a measure of the material’s toughness [2,11,49]. Much like the UCS and *E*_50_, the development of peak strain energy (or toughness) was found to be positively-proportional to the PC and/or FA contents, as well as the curing time (see Figure 4). The samples *P*_4_*F*_0_*T*_14_ and *P*_4_*F*_2_*T*_14_, for instance, resulted in peak strain energies of *E*_U_ = 4.0 kPa and 6.3 kPa, while the mix designs *P*_5_*F*_0_*T*_14_, *P*_5_*F*_2_*T*_14_, *P*_4_*F*_0_*T*_28_ and *P*_4_*F*_2_*T*_28_ resulted in *E*_U_ = 4.6, 7.4, 4.9 and 7.6 kPa, respectively.

**Figure 5:**
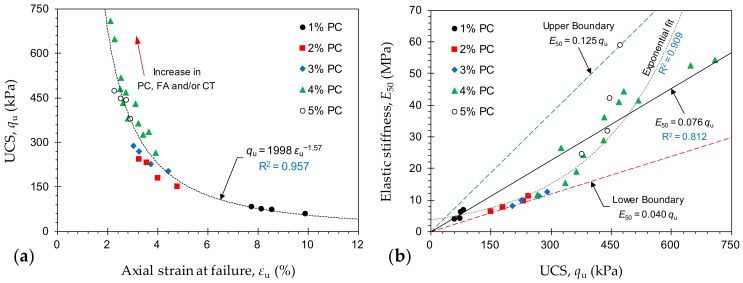
(**a**) Variations of UCS against axial strain at failure *ε*_u_—obtained by the external LVDTs—for the tested mix designs (CT = curing time); and (**b**) Variations of *E*_50_ against UCS for the tested mix designs.

The axial strain at failure, denoted as *ε*_u_ and obtained by the external LVDTs, is an indication of the material’s ductility; lower *ε*_u_ values manifest a more brittle (or less ductile) character [2,39]. Figure 5a illustrates the variations of UCS against *ε*_u_ for the tested samples. The greater the UCS, the lower the ductility, following a monotonically-decreasing trend—*q*_u_ = 1998*ε*_u_^−1.57^ (with R^2^ = 0.957). As such, the developed ductility was inversely-proportional to the PC and/or FA contents, as well as the curing time. The greater the PC and/or FA contents, the lower the axial strain at failure and hence the more brittle the sample failures.

Figure 5b illustrates the variations of *E*_50_ against *q*_u_ for the tested mix designs. The variations of *E*_50_ were situated within the 0.04*q*_u_ < *E*_50_ < 0.125*q*_u_ domain (*E*_50_ in MPa, and *q*_u_ in kPa). Moreover, *E*_50_ exhibited a rather strong correlation with the UCS. In this regard, simple correlative models in the forms of *E*_50_ = 0.076*q*_u_ (with R^2^ = 0.812) and *E*_50_ = 3.72 exp (4.7 × 10^−3^ *q*_u_) (with R^2^ = 0.909) can be derived, and thus implemented for indirect estimations of *E*_50_.

In the presence of water, PC initiates a series of chemical reactions in the tailings–water medium, which alter the composite’s fabric into a coherent matrix of enhanced strength attributes. The primary reactions include hydration of calcium silicates and calcium aluminates, both major components of PC, with water, thereby leading to the formation and propagation of strong cementation products/gels—calcium–silicate–hydrates (C-S-H) and calcium–aluminate–hydrates (C-A-H)—which encourage the development of a uniform, dense matrix coupled with enhanced strength and stiffness performance [19,38]. In this case, the greater the PC content, the greater the number of developed cementation products and hence the higher the mobilized strength and stiffness (e.g., see 0% FA in Figure 2a and Figure 3a). It should be noted that the primary reactions occur almost independently of the nature of the host material (or in this case tailings).

A byproduct of PC hydration is calcium hydroxide or Ca(OH)_2_, which promotes secondary reactions with any pozzolan agents present in the host material; the amount of released Ca(OH)_2_ is approximately 30% of the mass of the added PC [38]. Pozzolanic reactions are strongly time- and often temperature-dependent. During pozzolanic reactions, calcium cations (Ca^2+^) and hydroxide anions (OH^−^), both byproducts of the hydration stage, gradually react with silicate (SiO_2_) and aluminate (Al_2_O_3_) units from the tailings, thus producing additional C-S-H, C-A-H and C-A-S-H products in the matrix; these cementation products encourage flocculation and/or aggregation of the tailings particles and hence a further increase in the composite’s UCS and stiffness over time (e.g., see Figure 2b and Figure 3b) [2,11,37]. Quite clearly, the commencement and evolution of pozzolanic reactions is strongly dependent on the amount of released Ca(OH)_2_ (or the PC content), as well as the amount of available silicate and aluminate units (or pozzolan materials) in the matrix.

The addition of FA alongside PC induces the development of pozzolanic reactions, and hence cementation products, in the matrix through two potential pathways. As mentioned in Section 2.2, the Class C FA used in this study contains a notable fraction of calcium oxide (CaO). In the presence of water, these CaO units can be ionized to Ca^2+^ and OH^−^, thereby reacting with the remaining silicate and aluminate units in the tailings, as well as those present in FA itself, which in turn result in the development of additional cementation products and hence an increased strength and stiffness. Moreover, the Ca^2+^ and OH^−^ ions, formed through the ionization of Ca(OH)_2_ after the hydration stage, may also react with FA’s silicate and aluminate units, and thus encourage additional pozzolanic reactions over time. Accordingly, these additional cementation products lead to an improved mobilized strength and stiffness compared with similar PC inclusions containing no FA. As is evident from Figure 2a,b, for any given PC content, the developed strength, stiffness and toughness were consistently in favor of the FA content.

### 3.2. Field Strain Patterns

**Figure 6:**
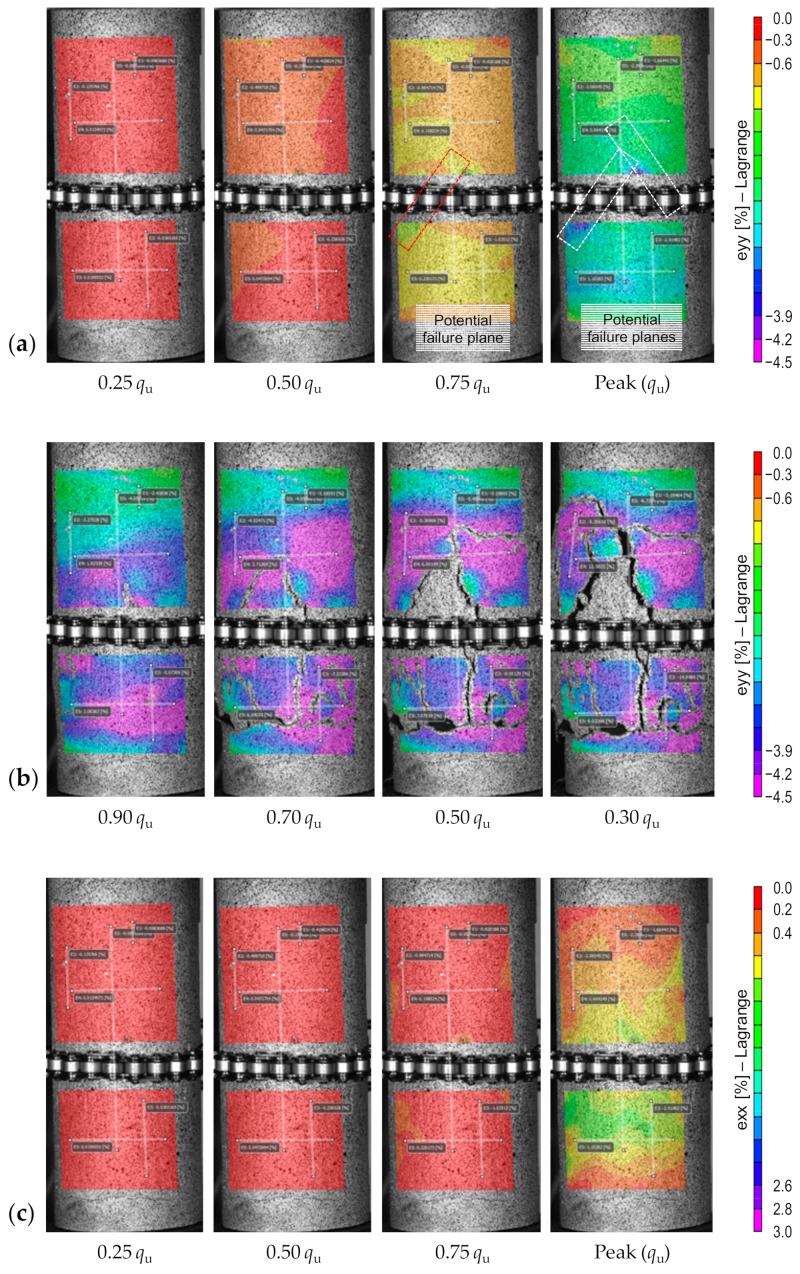

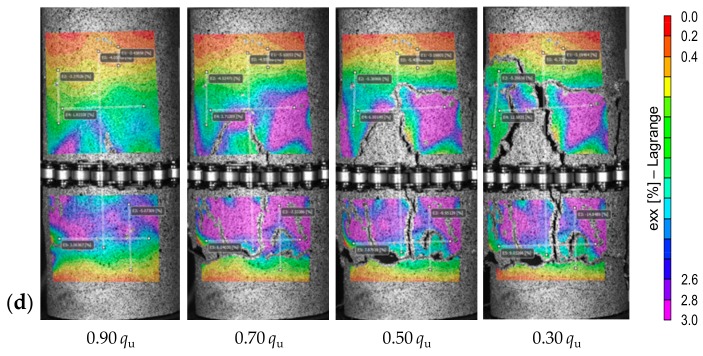
Field of strains developed at various axial stress levels for the sample *P*_4_*F*_0_*T*_14_: (**a**) Axial pre-peak; (**b**) Axial post-peak; (**c**) Lateral pre-peak; and (**d**) Lateral post-peak.

Figure 6a,b illustrates the field of axial strains developed for the sample *P*_4_*F*_0_*T*_14_ at various axial stress levels in the pre- and post-peak regimes, respectively. As outlined in Figure 6a, a total of four axial stress levels (i.e., 0.25*q*_u_, 0.5*q*_u_, 0.75*q*_u_ and *q*_u_ or the UCS), were considered to investigate the field of axial strain development during the pre-peak regime. The field of axial strains developed at 0.25*q*_u_, 0.5*q*_u_ and the most part of 0.75*q*_u_ each exhibited a rather uniform color pattern, thus implying that, up to 0.75*q*_u_, the sample undergoes uniform deformation during compressive loading. According to the CDZ crack framework, the failure process in quasi-brittle materials, and rocks in particular, under compressive loading takes place within an LDZ, where distributed cracking accompanied by a single major crack (i.e., single shear failure) or multiple major cracks (i.e., splitting failure) take place [26,27,28]. In this framework, the stress–strain response in the pre-peak regime describes the compressive behavior of the material in the entire sample [29]. Upon achieving the peak stress (or *q*_u_), accumulated large deformations take place (during unloading) outside of the damage zone; this is referred to as strain localization [30,31,32]. Theoretically, the behavior of CPB samples, particularly those containing high binder contents, should be consistent with the CDZ framework. However, as the CPB samples tested in this study contain low PC (or PC + FA) contents, their stress–strain evolution may be different from that of conventional quasi-brittle materials such as rock and concrete. As is evident from Figure 6a, the CPB sample *P*_4_*F*_0_*T*_14_ complies well with the CDZ framework up to 0.75*q*_u_, beyond which strain localization gradually takes place within the LDZ; at 0.75*q*_u_, the slight yellow-to-green color pattern, which represents an increase in strain density, signifies a linear plane of LDZ and hence a potential shear failure zone. As the axial stress approaches the UCS (or *q*_u_), the color gradient transitions to a non-uniform, blue-to-purple pattern at some locations, thereby indicating a further increase in strain density and hence the presence of multiple potential failure planes. Upon achieving the UCS, the axial stress begins to drop, and the sample’s character progressively transitions towards a strain-softening behavior. As outlined in Figure 6b, a total of four axial stress levels—0.9*q*_u_, 0.7*q*_u_, 0.5*q*_u_ and 0.3*q*_u_—were considered to investigate the field of axial strain development during the post-peak regime. The field of axial strains developed at these four stress levels all exhibited a non-uniform, blue-to-purple color pattern, thereby implying that the sample undergoes non-uniform, permanent deformation within the entire unloading domain. Moreover, normal shear faulting, which occurs in rock-like samples, was not observed in the CPB sample; instead, multiple large cracks—various vertical and horizontal shear bands—were found to evolve at different locations.

Figure 6c,d illustrates the field of lateral strains developed for the sample *P*_4_*F*_0_*T*_14_ at various axial stress levels in the pre- and post-peak regimes, respectively. Much like the field of axial strains shown in Figure 6a, the field of lateral strains developed during the pre-peak regime manifested a rather uniform color pattern up to 0.75*q*_u_, thereby indicating that the sample undergoes uniform deformation with no major signs of strain localization (see Figure 6c). A visual comparison between Figure 6b,d indicates that the locations of the LDZs identified by means of the post-peak lateral strain patterns (i.e., Figure 6d) are generally identical to those identified using their post-peak axial strain pattern counterparts (i.e., Figure 6b). It should be noted that these similarities between the developed field of axial and lateral strains were consistently observed for all of the tested samples during the pre- and post-peak regimes; therefore, additional contour graphs demonstrating the field of lateral strain development will not be presented. Moreover, the FA inclusions, under the current experimental design (see Table 4), did not alter the CPB’s failure pattern, as well as its field strain development in the pre- and post-peak regimes; for various mix designs tested at 14 days of curing (e.g., *P*_4_*F_b_T*_14_ where *b* = {0, 1, 2, 3}), the same unevenly-distributed splitting failure pattern was noted. On the contrary, the variables “PC content” and “curing time” were found to have some notable impacts on the field strain development. As such, to accommodate a more concise presentation of the experimental data, only the effects of PC content and curing time on strain localization will be discussed in the following.

**Figure 7:**
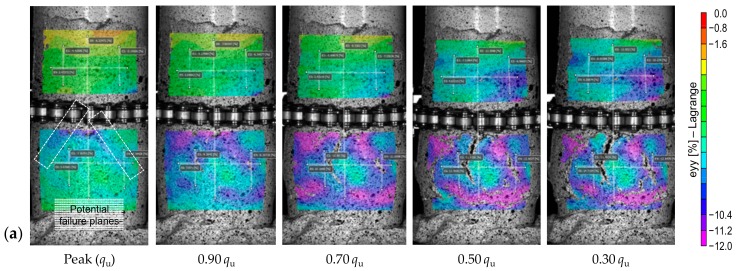

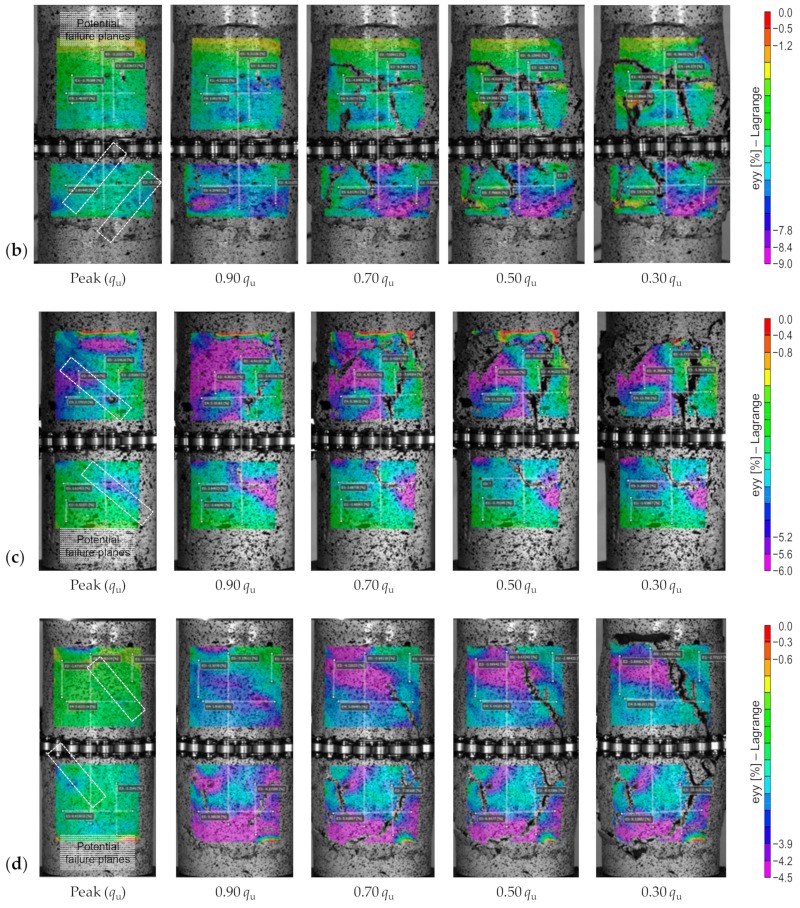
Effect of PC content on strain localization—field of axial strains developed at various post-peak axial stress levels: (**a**) *P*_1_*F*_0_*T*_14_; (**b**) *P*_2_*F*_0_*T*_14_; (**c**) *P*_3_*F*_0_*T*_14_; and (**d**) *P*_5_*F*_0_*T*_14_.

Figure 7 illustrates the effects of PC content—*P_a_F*_0_*T*_14_ where *a* = {1, 2, 3, 5}—on the field of axial strains developed at various axial stress levels in the post-peak regime (i.e., *q*_u_, 0.9*q*_u_, 0.7*q*_u_, 0.5*q*_u_ and 0.3*q*_u_. Much like the results obtained for the sample *P*_4_*F*_0_*T*_14_ in the pre-peak regime (see Figure 6a), strain localization in the samples containing 1%, 2%, 3% and 5% PC was found to initiate prior to, or immediately after, achieving the UCS (or *q*_u_). As is evident from Figure 7, the samples *P*_1_*F*_0_*T*_14_, *P*_2_*F*_0_*T*_14_, *P*_3_*F*_0_*T*_14_ and *P*_5_*F*_0_*T*_14_ did not exhibit normal shear faulting in the post-peak regime; instead, the failure process involved the evolution of multiple vertical and horizontal shear bands at different locations, which is consistent with that observed for the sample *P*_4_*F*_0_*T*_14_ (see Figure 6b). For the sample *P*_1_*F*_0_*T*_14_, the concentrated blue-to-purple color pattern, first encountered at the UCS, increased progressively (as accumulated large deformations transpire at the LDZ), and thus signifies future failure planes (see Figure 7a). As a result of 2% PC inclusion, however, strain localization was found to transpire with a rather irregular shape, and as such, potential failure planes could not be identified with confidence (see Figure 7b). In Figure 7c,d (i.e., *P*_3_*F*_0_*T*_14_ and *P*_5_*F*_0_*T*_14_), the blue and purple color patterns at the UCS indicate multiple potential failure zones, which eventually led to multiple fracturing. As discussed in Section 3.1, for any given curing time, the greater the PC content, the higher the developed UCS, following a monotonically-increasing trend. With an increase in PC content, and hence the CPB’s strength, the failure pattern gradually transitions from “multiple extension,” where multiple parallel fractures occurred along the axial direction (see *P*_1_*F*_0_*T*_14_ in Figure 7a), to “multiple fracturing,” where sample disintegration occurred in the form of several planes at various angles (e.g., see *P*_4_*F*_0_*T*_14_ in Figure 6b, and *P*_5_*F*_0_*T*_14_ in Figure 7d).

**Figure 8:**
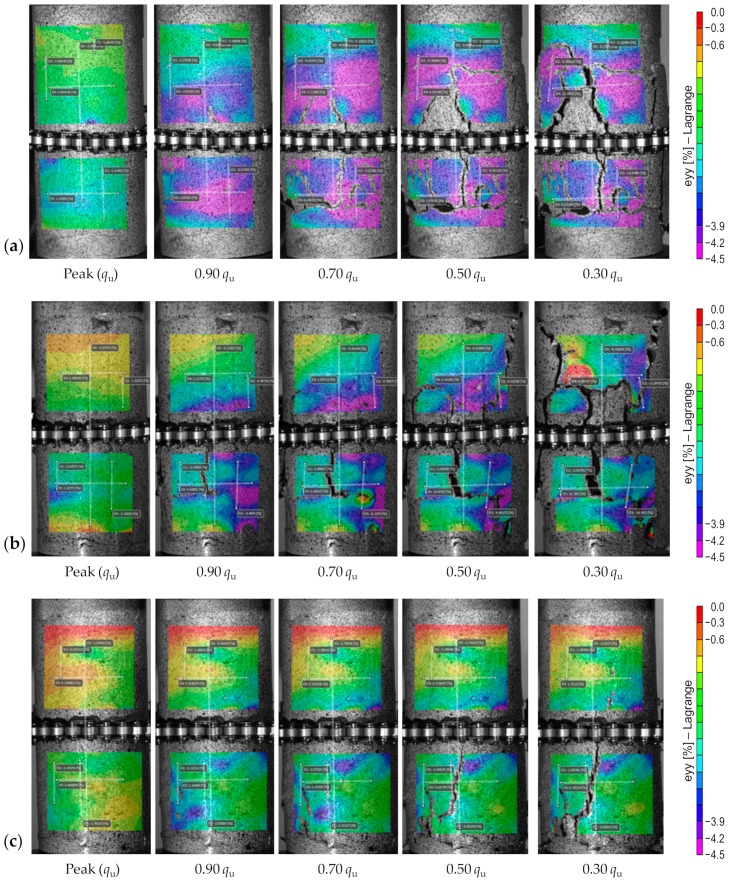
Effect of curing time on strain localization—field of axial strains developed at various post-peak axial stress levels: (**a**) *P*_4_*F*_0_*T*_14_; (**b**) *P*_4_*F*_0_*T*_28_; and (**c**) *P*_4_*F*_0_*T*_56_.

Figure 8 illustrates the effects of curing time—*P*_4_*F*_0_*T_c_* where *c* = {14, 28, 56}—on the field of axial strains developed at various axial stress levels in the post-peak regime (i.e., *q*_u_, 0.9*q*_u_, 0.7*q*_u_, 0.5*q*_u_ and 0.3*q*_u_. As a result of curing, the failure pattern was found to gradually transition towards a shear faulting (or single shear) character; for any given PC (or PC + FA) content, the longer the curing period, the more pronounced the shear faulting effect. Much like the sample cured for 14 days (or *P*_4_*F*_0_*T*_14_), the sample cured for 28 days (or *P*_4_*F*_0_*T*_28_) exhibited some blue color patterns at the UCS, which signify potential failure zones (compare Figure 8a,b at *q*_u_). In the post-peak regime, irregular strain localization was noted on the surface of both samples; this was perceived from the purple-to-pink color gradients observed in the areas of interest corresponding to the axial strains (e.g., see Figure 8a,b at 0.9*q*_u_). A visual comparison between the samples *P*_4_*F*_0_*T*_14_ and *P*_4_*F*_0_*T*_28_ also indicated that the LDZ reduced in size due to extended curing. Moreover, the failure pattern transitioned from “multiple fracturing” at 14 days of curing to “multiple/double shear” at 28 days of curing, where two large X-shaped shear bands, along with some irregular micro-cracks, transpired on the surface of *P*_4_*F*_0_*T*_28_. Unlike the samples cured for 14 and 28 days, the sample cured for 56 days (or *P*_4_*F*_0_*T*_56_) manifested a thin purple region in the post-peak regime, which represents an inclined plane of LDZ (see Figure 8c). At 56 days of curing, which is often sufficient for binder hydration, the failure pattern transitions to a clearly-visible single shear (or shear faulting) character, accompanied by some minor cracks; this failure pattern is rather similar to that reported for rock-like materials [50]. As such, the greater the PC content, and more importantly the longer the curing period, the more consistent the stress–strain evolution with that of conventional quasi-brittle materials such as rock and concrete.

### 3.3. Localization of the Stress–Strain Curves

**Figure 9:**
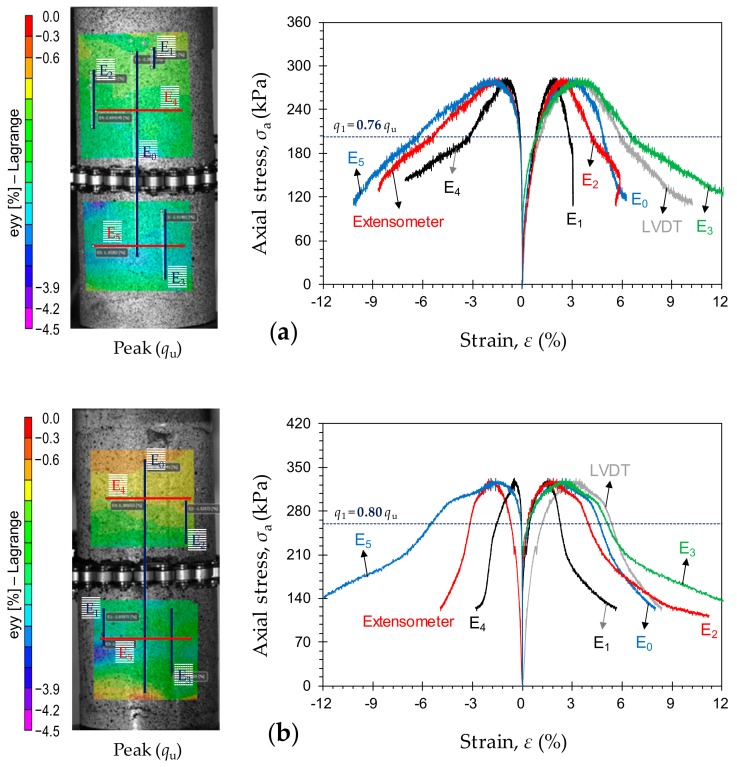

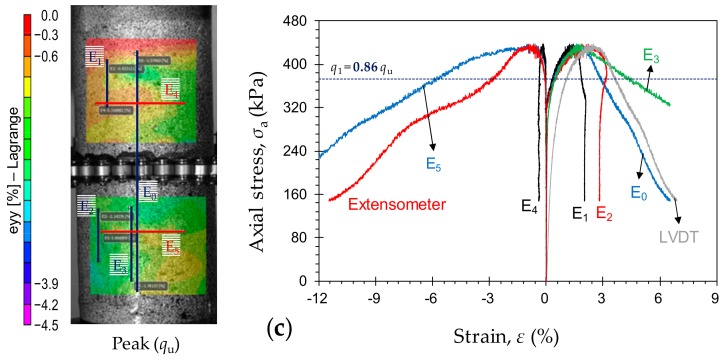
Typical stress–strain curves, obtained by means of various measurement techniques, for the tested samples: (**a**) *P*_4_*F*_0_*T*_14_; (**b**) *P*_4_*F*_0_*T*_28_; and (**c**) *P*_4_*F*_0_*T*_56_.

Typical stress–strain curves, obtained by means of various measurement techniques, for the samples *P*_4_*F*_0_*T*_14_, *P*_4_*F*_0_*T*_28_ and *P*_4_*F*_0_*T*_56_ are provided in Figure 9a–c, respectively. The axial and lateral strains, as outlined in the figures, were obtained by two different measurement techniques: (i) external measurement devices—the average of the two LVDTs for axial strain (see Figure 1c), and the direct-contact chain extensometer for lateral strain (see Figure 1c); and (ii) three-dimensional DIC. Using the VIC-3D software, four virtual axial extensometers (i.e., E_0_, E_1_, E_2_ and E_3_), as well as two virtual lateral extensometers (i.e., E_4_ and E_5_), were appointed to measure the local stress–strain characteristics; the locations of these virtual extensometers are shown in Figure 9. The extensometer E_0_ captures the axial strains along the entire sample, thus representing the overall average axial deformation (comparable to the external LVDTs) during compressive loading. The extensometer E_1_ measures the axial strains outside of the LDZ, while E_2_ and E_3_ capture the axial strains in regions where strain localization takes place. Similarly, the irreversible lateral strains were measured by means of two local virtual extensometers, E_4_ and E_5_, which, respectively, measure the lateral strains outside and inside of the LDZ. As is evident from Figure 9, the axial strains measured by E_0_ displayed a trend similar to that captured by the LVDTs. Much like E_0_, the LVDTs represent the overall average axial deformation along the entire sample, however, with a certain level of contained bedding error. In contrast, strain measurements by means of the DIC technique are free from bedding errors [29,51,52,53]. Given that the bedding error varies from sample to sample, the overall average axial strain recorded by the DIC technique (i.e., E_0_), can be employed to describe the stress–strain attributes of CPB samples with an increased degree of accuracy. For any given CPB sample (e.g., *P*_4_*F*_0_*T*_14_) the stress–strain curves in the pre-peak regime—LVDT, E_0_, E_1_, E_2_ and E_3_, as shown in Figure 9a—were found to perfectly overlap with each other (up to approximately 0.8*q*_u_), thus indicating that the sample undergoes uniform axial deformation with no major signs of strain localization. Similarly, the lateral strains generated by the external chain extensometer, E_4_ and E_5_, were rather identical up to approximately 80% of the UCS (e.g., see Figure 9a).

As is evident from Figure 9, for any given PC (or PC + FA) content, an increase in curing time led to a decrease in the axial strain localization behavior during the pre-peak regime. The sample *P*_4_*F*_0_*T*_14_ achieved its UCS at 2.49%, 3.11% and 3.46% based on E_1_, E_0_ and E_2_, respectively (see Figure 9a). After the UCS, however, strain localization takes place, and the three virtual extensometers generate different values with notable differences. For further reference, the axial strain at the UCS, based on E_1_, E_0_ and E_2_, was measured as 2.05%, 2.45% and 2.63% for *P*_4_*F*_0_*T*_28_, and 1.31%, 1.46% and 1.67% for *P*_4_*F*_0_*T*_56_, respectively (see Figure 9b,c). In the pre-peak regime, the effect of curing time was found to be positively-proportional to the axial stress level required to initiate localization. As demonstrated in Figure 9, the axial stress at which localization initiates can be taken as *q*_1_ = 202.5 kPa, 259.5 kPa and 372.8 kPa for the samples *P*_4_*F*_0_*T*_14_, *P*_4_*F*_0_*T*_28_ and *P*_4_*F*_0_*T*_56_, respectively. Taking into account the samples’ UCS values, i.e., *q*_u_ = 265.3, 325.2 and 432.7 kPa, the axial stress level required to initiate localization can be calculated as 0.76*q*_u_, 0.8*q*_u_ and 0.86*q*_u_, respectively. In essence, as the curing time increases, the axial stress level required to initiate localization approaches the UCS, thus emulating the failure mechanism of *quasi-brittle* materials such as rock and concrete [31,32]. Unlike the pre-peak strains, for any given PC (or PC + FA) content, the post-peak axial and lateral strains exhibited a more pronounced strain localization effect with an increase in curing time; in this regard, the effect of curing was found to be more dominant on the lateral strain localization (e.g., compare the post-peak axial and lateral strains in Figure 9a,c). For the sample *P*_4_*F*_0_*T*_56_, as shown in Figure 9c, the virtual extensometers E_1_, E_2_ and E_4_ (located outside of the LDZ where shear failure takes place) experienced inelastic unloading, and the strain rarely increased after the UCS. Finally, with an increase in curing time, the difference between strain values at the localized and non-localized zones becomes less significant in the pre-peak regime and more pronounced in the post-peak regime.

**Figure 10:**
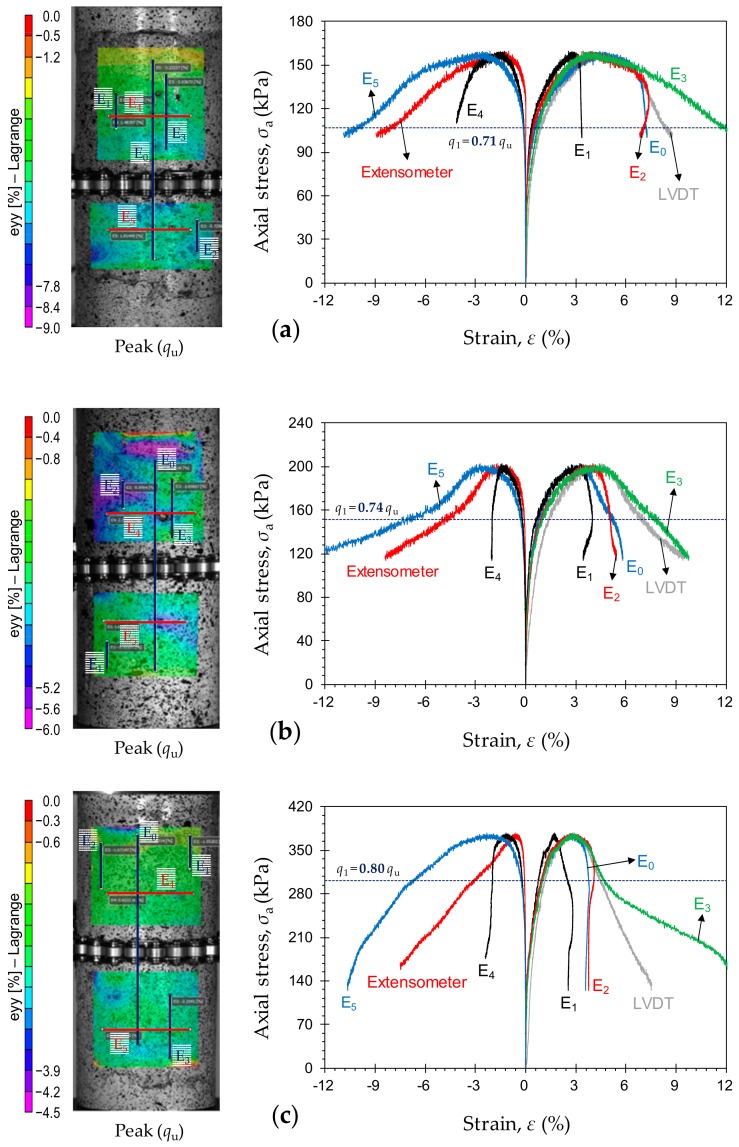
Typical stress–strain curves, obtained by means of various measurement techniques, for the tested samples: (**a**) *P*_2_*F*_0_*T*_14_; (**b**) *P*_3_*F*_0_*T*_14_; and (**c**) *P*_5_*F*_0_*T*_14_.

Typical stress–strain curves, obtained by means of various measurement techniques, for the samples *P*_2_*F*_0_*T*_14_, *P*_3_*F*_0_*T*_14_ and *P*_5_*F*_0_*T*_14_ are provided in Figure 10a–c, respectively. At any given curing time, an increase in PC content resulted in a decrease in the axial strain localization behavior during the pre-peak regime. This effect, however, was slightly less pronounced compared with that imposed by an increase in curing time. Much like the effect of curing time, the effect of PC content was found to be positively-proportional to the axial stress level required to initiate localization. At any given curing time, the greater the PC content, the closer the axial stress level required to initiate localization (or *q*_1_) to the UCS (*q*_u_), and as such, the more similar the failure mechanism of the CPB sample to that of rock and concrete. For instance, the axial stress level required to initiate localization was obtained as *q*_1_ = 0.71*q*_u_, 0.74*q*_u_ and 0.80*q*_u_ for the samples *P*_2_*F*_0_*T*_14_, *P*_3_*F*_0_*T*_14_ and *P*_5_*F*_0_*T*_14_, respectively (see Figure 10).

**Figure 11:**
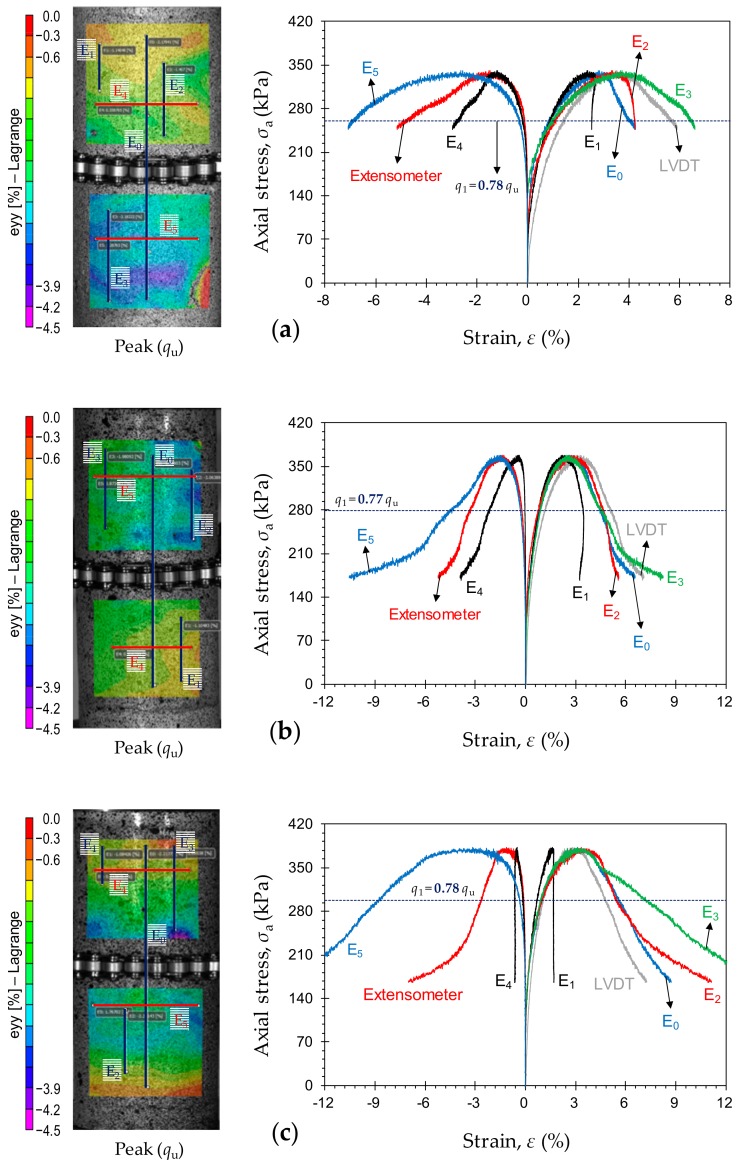
Typical stress–strain curves, obtained by means of various measurement techniques, for the tested samples: (**a**) *P*_4_*F*_1_*T*_14_; (**b**) *P*_4_*F*_2_*T*_14_; and (**c**) *P*_4_*F*_3_*T*_14_.

Typical stress–strain curves, obtained by means of various measurement techniques, for the samples *P*_4_*F*_1_*T*_14_, *P*_4_*F*_2_*T*_14_ and *P*_4_*F*_3_*T*_14_ are provided in Figure 11a–c, respectively. Unlike curing time and PC content, FA content did not significantly alter the localization behavior. At any given curing time, and for any given PC content, the axial stress level required to initiate localization (or *q*_1_) was found to be rather independent of the FA content. For instance, as demonstrated in Figure 11, the axial stress level required to initiate localization for the samples *P*_4_*F*_1_*T*_14_, *P*_4_*F*_2_*T*_14_ and *P*_4_*F*_3_*T*_14_ can be, respectively, calculated as *q*_1_ = 0.78*q*_u_, 0.77*q*_u_ and 0.78*q*_u_; these values are nearly equal to that obtained for the same 4% PC cured for 14 days containing no FA (i.e., *P*_4_*F*_0_*T*_14_ with *q*_1_ = 0.76*q*_u_, as outlined in Figure 9a).

## 4. Concluding Remarks

The present study has arrived at the following conclusions.

The greater the PC content and/or the longer the curing period, the higher the developed strength, stiffness and toughness, with the former, PC content, portraying a more significant role. The axial strain at failure—an indication of the material’s ductility—demonstrated a trend similar to that observed for strength and stiffness; however, in an adverse manner. The use of FA alongside PC led to further strength and stiffness improvements by way of inducing secondary pozzolanic reactions. Common strength criteria for CPBs were considered to assess the applicability of the tested PC + FA mix designs; with regards to stope stability, for instance, the sample *P*_4_*F*_3_*T*_56_ was found to satisfy the minimum 700 kPa threshold, and thus was deemed as the optimum choice.

As opposed to external measurement devices, the DIC technique was found to provide strain measurements free from bedding errors. The developed field of axial and lateral strains indicated that strain localization initiates in the pre-peak regime at around 80% of the UCS. The greater the PC (or PC + FA) content, and more importantly, the longer the curing period, the closer the axial stress level required to initiate localization to the UCS, thus emulating the failure mechanism of quasi-brittle materials such as rock and concrete; the failure process changed from “multiple fractures parallel and perpendicular to the axial loading direction” to “sample disintegration” where a single major failure plane became noticeable. For instance, the axial stress level required to initiate localization increased from 0.76*q*_u_ for *P*_4_*F*_0_*T*_14_ to 0.86*q*_u_ for *P*_4_*F*_0_*T*_56_. Finally, with an increase in curing time, the difference between strain values at the localized and non-localized zones became less significant in the pre-peak regime and more pronounced in the post-peak regime.

## Figures and Tables

**Table 1:** Geotechnical properties and chemical composition of the mine tailings.

Geotechnical Properties	Standard	Value	Chemical Components	MF (%) ^1^
Specific gravity, *G*_s_	ASTM D854–14	2.61	SiO_2_	38.27
Fines content (<75 μm) (%)	ASTM D422–07	39	Fe_2_O_3_	37.70
Sand content (0.075–2 mm) (%)	ASTM D422–07	61	Al_2_O_3_	7.19
Liquid limit, LL (%)	AS 1289.3.9.1–15	19.2	K_2_O	2.33
Plastic limit, PL (%)	AS 1289.3.2.1–09	13.1	CaO	0.81
Plasticity index, PI (%) ^2^	AS 1289.3.3.1–09	6.1	MgO	0.75
USCS classification ^3^	ASTM D2487–11	CL–ML ^4^	TiO_2_	0.56
Optimum water content (%)	ASTM D698–12	8.7	Na_2_O	0.07
Maximum dry density (g/cm^3^)	ASTM D698–12	2.06	Other	12.32

^1^ Mass fraction; ^2^ PI = LL − PL; ^3^ Unified Soil Classification System; and ^4^ Clay–silt with low plasticity.

**Table 2:** Physical and chemical properties of PC.

Properties	Value	Standard
Fineness index (m^2^/g)	370–430	AS 2350.8–06
Initial setting time (min)	105	AS 2350.4–06
Final setting time (min)	180	AS 2350.4–06
3-Day compressive strength (MPa)	31	AS 2350.11–06
7-Day compressive strength (MPa)	42	AS 2350.11–06
28-Day compressive strength (MPa)	60	AS 2350.11–06
Portland clinker (% by mass)	85–93	–
Natural gypsum (% by mass)	5–7	–
Mineral additives (% by mass)	<8	–
Sulfur trioxide, SO_3_ (% by mass)	2.8	–
Equivalent alkalis (% by mass)	0.5	–
Chloride, Cl^−^ (% by mass)	0.05	–
Loss on ignition, LOI (at 1000 °C) (%)	3–4	–

**Table 3:** Chemical composition of the processed mine water.

Chemical Components	Concentration (mg/L)
Chloride, Cl^−^	5800
Sodium, Na^+^	3800
Sulfate, SO_4_^2−^	2400
Calcium, Ca^2+^	480
Potassium, K^+^	380
Magnesium, Mg^2+^	280
Nitrate, NO_3_^−^	6

**Table 4:** Mix designs and their properties.

Designation	PC (%) ^1^	FA (%) ^1^	SC (%) ^2^	WC (%) ^3^	Tests
*P*_1_ *F*_0_ *T*_14_	1	0	77	30	UCS ^4^ + DIC ^5^
*P*_1_ *F*_1_ *T*_14_	1	1	77	30	
*P*_1_ *F*_2_ *T*_14_	1	2	77	30	
*P*_1_ *F*_3_ *T*_14_	1	3	77	30	
*P*_2_ *F*_0_ *T*_14_	2	0	77	30	UCS + DIC
*P*_2_ *F*_1_ *T*_14_	2	1	77	30	
*P*_2_ *F*_2_ *T*_14_	2	2	77	30	
*P*_2_ *F*_3_ *T*_14_	2	3	77	30	
*P*_3_ *F*_0_ *T*_14_	3	0	77	30	UCS + DIC
*P*_3_ *F*_1_ *T*_14_	3	1	77	30	
*P*_3_ *F*_2_ *T*_14_	3	2	77	30	
*P*_3_ *F*_3_ *T*_14_	3	3	77	30	
*P*_4_ *F*_0_ *T*_14,28,56_	4	0	77	30	UCS + DIC
*P*_4_ *F*_1_ *T*_14,28,56_	4	1	77	30	
*P*_4_ *F*_2_ *T*_14,28,56_	4	2	77	30	
*P*_4_ *F*_3_ *T*_14,28,56_	4	3	77	30	
*P*_5_ *F*_0_ *T*_14_	5	0	77	30	UCS + DIC
*P*_5_ *F*_1_ *T*_14_	5	1	77	30	
*P*_5_ *F*_2_ *T*_14_	5	2	77	30	
*P*_5_ *F*_3_ *T*_14_	5	3	77	30	

^1^ Equation (2); ^2^ Equation (3); ^3^ Equation (4); ^4^ Unconfined compressive strength; and ^5^ Digital image correlation.

**Table 5:** Strength criteria for CPBs adopted in mining applications (modified from [11]).

Applications	UCS, *q*_u_ (kPa)	Reference
Roof support	>4000	[41,42]
Stope stability	700–2000	[43]
Surface disposal	≥345	[44]
General construction practices	≥345	[11,45]
Eliminating liquefaction	150–300	[42,46]

## Abbreviations

The following abbreviations are used in this manuscript.

AS	Australian Standard
ASTM	American Society for Testing and Materials
BC	Binder Content
C-A-H	Calcium–Aluminate–Hydrate
C-A-S-H	Calcium–Aluminate–Silicate–Hydrate
CDZ	Crack Damage Zone
CL	Clay with Low Plasticity
CLSM	Controlled Low-Strength Material
CPB	Cemented Paste Backfill
C-S-H	Calcium–Silicate–Hydrate
CT	Curing Time
DIC	Digital Image Correlation
FA	Fly Ash
LDZ	Local Damage Zone
LL	Liquid Limit
LVDT	Linear Variable Differential Transducer
ML	Silt with Low Plasticity
PC	Portland Cement
PI	Plasticity Index
PL	Plastic Limit
SC	Solids Content
UCS	Unconfined Compressive Strength
USCS	Unified Soil Classification System
WC	Water Content
